# Nutraceuticals and Bioactive Components from Fish for Dyslipidemia and Cardiovascular Risk Reduction

**DOI:** 10.3390/md14060113

**Published:** 2016-06-08

**Authors:** Giulia Chiesa, Marco Busnelli, Stefano Manzini, Cinzia Parolini

**Affiliations:** Department of Pharmacological and Biomolecular Sciences, Università degli Studi di Milano, via Balzaretti 9, Milano 20133, Italy; giulia.chiesa@unimi.it (G.C.); marco.busnelli@gmail.com (M.B.); stefano.manzini@gmail.com (S.M.)

**Keywords:** cardiovascular disease, clinical trials, cholesterol, fish proteins, hypertension, *n*-3 polyunsaturated fatty acids, triglycerides

## Abstract

Cardiovascular disease remains the most common health problem in developed countries, and residual risk after implementing all current therapies is still high. Permanent changes in lifestyle may be hard to achieve and people may not always be motivated enough to make the recommended modifications. Emerging research has explored the application of natural food-based strategies in disease management. In recent years, much focus has been placed on the beneficial effects of fish consumption. Many of the positive effects of fish consumption on dyslipidemia and heart diseases have been attributed to *n*-3 polyunsaturated fatty acids (*n*-3 PUFAs, *i.e.*, EPA and DHA); however, fish is also an excellent source of protein and, recently, fish protein hydrolysates containing bioactive peptides have shown promising activities for the prevention/management of cardiovascular disease and associated health complications. The present review will focus on *n*-3 PUFAs and bioactive peptides effects on cardiovascular disease risk factors. Moreover, since considerable controversy exists regarding the association between *n*-3 PUFAs and major cardiovascular endpoints, we have also reviewed the main clinical trials supporting or not this association.

## 1. Introduction

Cardiovascular disease (CVD) is the most common cause of human morbidity and mortality in the world, and elevated blood lipids have been strongly associated with increased incidence of CVD. Expenses for the treatment of CVD to the European health care system are presumed to be as high as 200 billion Euros each year [1]. In the USA, the overall rate of deaths attributed to CVD in 2011 was 229.6 per 100,000 Americans [2]. There are estimates that in 2030, nearly 23.6 million people will die from CVD worldwide [3]. Due to an aging population and shifting risks posed by the environment, this burden is expected to increase in developing countries.

Atherosclerosis is the dominant cause of CVD including myocardial infarction, heart failure, stroke and claudication. Atherosclerosis is mainly located in the intima of middle sized and large arteries, especially where the vessels divide [4,5]. It is well known that atherosclerosis develops during a long period of time so the earlier the lipid management is initiated the more likely the atherosclerotic vascular diseases can be prevented [6].

Lowering low density lipoprotein-cholesterol (LDL-C) is a central target in the prevention of CVD, especially coronary heart disease. It is estimated that for every 1% reduction in LDL-cholesterol concentration, there is a corresponding 1% to 2% decrease in the risk of CVD [7,8,9,10].

Although genetic factors and aging are important in determining the overall risk, a substantial proportion of CVD occurs in conjunction with a series of modifiable risk factors, such as hyperlipidemia, hypertension, obesity, insulin resistance and diabetes, susceptible to lifestyle modifications, which include diet and physical exercise [11,12]. Permanent changes in lifestyle may be hard to achieve, however, and people may not always be motivated enough to make the recommended changes [6]. One solution could be the consumption of foods, which favorably affect the above-mentioned risk factors, such as nutraceuticals. Nutraceutical is a term coined in 1979 by Stephen DeFelice and defines a “food, or parts of food, that provide medical or health benefits, including the prevention and treatment of disease” [13].

With marine species comprising approximately one-half of the global biodiversity, the ocean offers a wonderful resource for novel compounds, which may serve in improving health of the worldwide population.

Observational studies first reported in the 1970s showed that the Greenland Inuit populations had a low incidence of coronary artery disease that was related to their traditional lifestyle and in particular their distinctive dietary habits, characterized by the consumption of cold-water marine fish and artic mammals, rich in Ω-3 (or *n*-3) polyunsaturated fatty acids (PUFAs), particularly eicosapentaenoic acid (EPA, 20:5 *n*-3) and docosahexaenoic acid (DHA, 22:6 *n*-3) [14,15,16]. Subsequently, researchers from several prospective epidemiological studies reported that high fish consumption was associated with a lower mortality from coronary artery disease [17,18]. However, benefits of *n*-3 PUFAs intake were challenged by recent clinical trials that failed to replicate protective effects of EPA and DHA on CVD [19]. It is, therefore, possible that the potential benefit of fish consumption could, in addition to *n*-3 PUFAs, be attributed to other nutrients, such as minerals, vitamins and proteins [20]. Fish protein hydrolysates containing bioactive peptides have shown promising activities for the prevention/management of cardiovascular disease and associated health complications [21].

This review examines current recommendations for fish intake, as source of *n*-3 PUFAs and bioactive peptides, and their effects on risk factors for CVD and on specific clinical endpoints.

## 2. *n*-3 PUFAs and Cardiovascular Risk Factors

**Figure 1:**
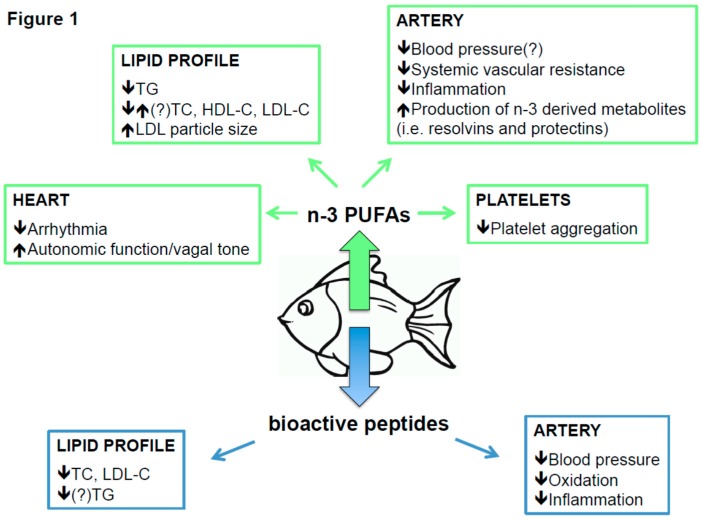
Beneficial effects of *n*-3 PUFAs and peptides from fish on cardiovascular risk factors. *n*-3 PUFAs: *n*-3 polyunsaturated fatty acids; TG: triglycerides; TC: total cholesterol; HDL-C: high density lipoprotein-cholesterol; LDL-C: low density lipoprotein-cholesterol.

Several data highlighted that *n*-3 PUFAs are able to affect: lipid profile [22,23,24], arrhythmia [25], platelet activity [26,27], endothelial function [28,29], inflammation [30], and blood pressure (Figure 1) [31].

### 2.1. n-3 PUFAs and Dyslipidemias

Fasting and nonfasting triglycerides (TG) have long been associated with CVD [32]. Fish oils rich in *n*-3 PUFAs have well known and long-appreciated TG-lowering properties [33]. Intake of 4 g of Lovaza [34], a drug formulation containing ethyl ester of EPA (465 mg) and DHA (375 mg), reduce serum TG by 20%–50%, depending on baseline values [33,35,36].

Recently, the EpanoVa fOr Lowering Very high triglyceridEs (EVOLVE) trial, a double-blind, randomized, controlled investigation of a lipid-altering drug in patients with severe hypertriglyceridemia, demonstrated that Epanova [37], a novel formulation containing the free fatty acid forms of both EPA and DHA, significantly lowers TG and non-high density lipoprotein-cholesterol (HDL-C) concentrations at all the tested doses, *i.e.*, 2, 3, and 4 g/day [36]. Specifically, Epanova at 2-, 3-, and 4-g/day dosages significantly reduced TG levels from baseline by 25.9%, 25.5%, and 30.9%, respectively, compared with a 4.3% decline in subjects taking 4 g/day of olive oil. It is important to underline that approximately 84% of the TG reduction at 4 g/day was already present at the 2-g/day dosage [38]. Since it has been demonstrated that, in individuals on a low-fat diet, bioavailability of EPA + DHA from Epanova (free fatty acid form of EPA + DHA) is four-fold higher than that from Lovaza (ethyl ester form of EPA + DHA), the authors hypothesized that the greater bioavailability may have enhanced the efficacy at lower dosages [38,39]. The explanation of the Epanova’s greater bioavailability may, at least in part, reside in the fact that, unlike the ethyl ester forms of *n*-3 PUFA, the free fatty acid form does not require pancreatic lipase hydrolysis with carboxyl ester lipase, an enzyme which activity is highly dependent on fat meal content [39]. It can then be speculated that absorption of free fatty acid form of EPA + DHA would not be compromised by a fat intake restriction and would offer a therapeutic advantage over the ethyl ester form in patients with severe hypertriglyceridemia.

The mechanisms by which *n*-3 PUFAs accomplish this hypotriglyceridemic effect have been explored in kinetic studies in humans [40,41] and animals [42,43], as well as in *in vitro* and *ex vivo* experiments [44]. The majority of the animal studies have been performed in rats and the results, even though are not always consistent, suggested that at least three mechanisms are responsible for the hypotriglyceridemic effect: (1) reduced fatty acids availability (due to inhibition of *de novo* lipogenesis, decrease of serum levels of nonesterified fatty acids and increase in fatty acid beta-oxidation); (2) reduced hepatic enzyme activity for TG synthesis; (3) increased phospholipid synthesis and apoB degradation [33,45,46]. Human studies have shown that *n*-3 PUFAs exert their effect on TG by inhibiting hepatic very low density lipoprotein (VLDL) secretion rate and by stimulating serum TG clearance mechanisms [47,48,49,50,51,52].

With regard to total cholesterol (TC), HDL-C and LDL-C levels, *n*-3 PUFAs have little or no effect on TC, but affect LDL-C and HDL-C concentrations as well as HDL and LDL particle size; these effects are variable and depend on dose and population studied [53,54,55,56]. Moreover, recent evidences have shown that there are individual differences between EPA and DHA [57]. DHA supplements increased HDL-C concentrations and LDL particle size [56], whereas a 4 g/day dose of Vascepa [58], a preparation of EPA as ethyl ester, significantly reduced TC, non-HDL-C and apoB plasma levels in hypertriglyceridemic patients without raising LDL-C concentrations [59]. Similar effects were observed in patients with metabolic syndrome [60].

Currently, six *n*-3 PUFA formulations are approved in the United States to treat adult people with very high levels of TG (>5.6 mmolL) with or without concomitant elevation of other atherogenic parameters: omega-3-acid ethyl esters (Lovaza, Omtryg, and 2 generic formulations), omega-3-carboxylic acids (Epanova), which contain both EPA and DHA, and icosapent ethyl (Vascepa), which is an EPA-only formulation [61].

### 2.2. n-3 PUFAs and Arrhythmia

About 80%–90% of sudden cardiac deaths in the early stages after a myocardial infarction are linked to ventricular arrhythmias, and arrhythmias are associated with electrophysiological mechanisms controlling muscle contraction [62]. *In vitro* and animal studies showed that *n*-3 PUFAs directly affect cardiac ion channels [62,63]. Data obtained with cardiomyocytes suggested that *n*-3 PUFAs exert a marked inhibitory effect on sodium channels, thus reducing excitability, and a wide range of effects on potassium channels, *L*-type calcium channels and sodium-calcium exchanger [62,64,65,66]. Moreover, *n*-3 PUFAs have been shown to alter membrane fluidity that could contribute to effects on ion transport [67,68]. Studies in dogs and in pigs showed that treatment with *n*-3 PUFAs reduces ischaemia-induced ventricular fibrillation, by acting on potassium channels [69,70]. Anti-arrhythmic effects of *n*-3 PUFAs may be mediated in part by their actions on autonomic control, especially by an increased vagal tone [71]. Altogether these mechanisms would be consistent with anti-arrhythmic effects and reduction of sudden cardiac death observed in at least some human studies [17,72,73,74,75].

### 2.3. n-3 PUFAs and Platelet Activity

*n*-3 PUFAs are considered to have anti-thrombotic effects due to their ability to inhibit platelet thromboxane A2 synthesis and to act as antagonists of the pro-aggregant thromboxane A2/prostaglandin H2 receptor [76]. However, these effects have been observed at very high doses (15 g/day) and for this reason in human trials no consistent effects on platelet aggregation or coagulation factors were detected [77].

### 2.4. n-3 PUFAs and Endothelial Function and Inflammation

The mechanism by which *n*-3 PUFAs influence endothelial function is mediated by their incorporation into biological membrane phospholipids; this allows modulation of membrane composition and fluidity [78]. Endothelial cell membrane houses caveolae and lipid rafts where several receptors and signaling molecules crucial for cell function are concentrated [79]. Caveolae associated receptor-mediated cellular signal transduction includes important pathways such as the nitric-oxide (NO) cGMP pathway, the NADPH oxidase and TNF-α-NFκB induced cyclooxygenase-2 and prostaglandin E2 activation pathway [80,81]. By modulating the composition of caveolae, as described for other lipid classes [82], *n*-3 PUFAs may exert their beneficial effects, which include increased NO production and reduced production of pro-inflammatory mediators. Molecular evidence of enhanced endothelial nitric oxide synthase activity/expression following administration of *n*-3 PUFAs derives from *in vitro* and *in vivo* experimental studies [83,84]. In endothelial cells, *n*-3 PUFAs attenuate NF-κB activation, resulting in reduced vascular cell adhesion molecule-1 expression [85,86]. Additionally, *n*-3 PUFAs exert systemic anti-inflammatory effects by raising the plasma levels of adiponectin [87] and suppressing the production of interleukin 6, interleukin 1β, soluble E selectin, and C-reactive protein [65]. Moreover, *n*-3 PUFAs are precursors of a novel series of lipid mediators (e.g., resolvins, protectins, and maresins) with potent anti-inflammatory and pro-resolving properties [88].

### 2.5. n-3 PUFAs and Blood Pressure

Several studies have indicated that relatively high doses of *n*-3 PUFAs are able to reduce blood pressure in both normotensive and hypertensive subjects, even though this effect was more pronounced in the latest group [31,89,90,91]. However, in a recent study, Minihane *et al.* have shown that intakes of EPA + DHA, achievable through the consumption of 2–3 portions of oily fish/week or 2 fish oil capsules/day, reduced systolic blood pressure by 5 mm Hg in isolated systolic hypertensive adult subjects [92]. This effect could be the result of the *n*-3 PUFAs ability to reduce thromboxane A2 synthesis, increase NO production, and affect the autonomic nerve function [54,93,94].

## 3. Clinical Trials with *n*-3 PUFAs: Past and Future

In the Diet and Reinfarction Trial (DART) study, 2033 men after myocardial infarction were randomly assigned to a group instructed to increase fish intake (corresponding to about 900 mg/day of EPA + DHA) or to a control group that received no specific information and followed for 2 years. The subjects advised to eat fatty fish had a 29% reduction in 2-year all-cause mortality compared with those not advised [95].

A double-blind, randomized, controlled trial was conducted, in which 205 patients undergoing a first percutaneous trans-luminal coronary angioplasty (PTCA) received 15 capsules per day containing 1 g of either fish oil (2.7 g/day of EPA and 1.8 g/day of DHA) or olive oil. The treatment was started 3 weeks before PTCA and continued for 6 months thereafter. Restenosis occurred significantly less often in the fish oil group (22.0%–35.6%) than in the control group (40.0%–53.3%) [96].

The Gruppo Italiano per lo Studio della Sopravvivenza nell’Infarto Miocardico (GISSI)-Prevenzione trial enrolled 11,324 patients with recent myocardial infarction and randomized them to the following treatment groups: vitamin E (300 mg/day), or EPA and DHA ethyl esters (very similar to Lovaza, 850–882 mg/day) or no supplements (usual care). After 3.5 years of follow-up, the group given *n*-3 PUFAs experienced a 15% reduction in the primary end point of death, nonfatal myocardial infarction, and nonfatal stroke (*p* < 0.02). Moreover, there was a 20% and a 45% reduction in all-cause of mortality and in sudden death, respectively [73].

The Japanese EPA Lipid Intervention Study (JELIS) investigated the effects of purified EPA for prevention of major coronary events [97]. A total of 18,645 Japanese individuals with hypercholesterolemia (TC ≥ 6.5 mmol/L) were randomized to receive 1.8 g/day EPA plus a statin (pravastatin or simvastatin) or statin only over 5 years. The risk of major coronary events was reduced by 19% in the EPA group compared with the statin-only group (*p* = 0.011) and by 53% in the sub-group with TG ≥ 150 mg/dL (1.70 mmol/L) and HDL-C < 40 mg/dL (1.04 mmol/L) (HR: 0.47; *p* = 0.043) [98].

The later GISSI-HF study (*N* = 7046) demonstrated a small (1.8%), but still significant, reduction for all-cause mortality in patients with clinical evidence of heart failure (60% of subjects had New York Class II symptoms, and 40% had previous myocardial infarction) who had been treated with 1 g/day of *n*-3 PUFAs in addition to 10 mg/day of rosuvastatin [99].

Patients with chronic heart failure due to nonischemic dilated cardiomyopathy and minimal symptoms while receiving evidence-based therapy were assessed prospectively by echocardiography at baseline and at 12 months after randomization to either 2 g of Lovaza or placebo. The main findings of this study were that 1-year treatment with *n*-3 PUFAs improves parameters of LV systolic and diastolic function, as well as functional capacity. However, this study had several limitations, *i.e.*, single-center trial, a small sample size and a limited number of clinical events [100].

While these earlier studies on CV outcomes reported favorable effects of *n*-3 PUFAs, subsequent clinical trials evaluating the EPA and DHA combination therapy have been disappointing.

A randomized, double-blind, placebo-controlled trial was performed at 6 US medical centers with enrollment from February 1999 until January 2003. Patients were randomly assigned to receive fish oil, 1.8 g/day, consisting of 42% EPA and 30% DHA as ethyl esters, or placebo and were followed up for two years. Among patients with a recent episode of sustained ventricular arrhythmia and an implantable cardioverter defibrillator, fish oil supplementation does not reduce the risk of ventricular tachycardia or ventricular fibrillation and may be proarrhythmic in some patients [101]. Three double-blind, randomized intervention studies in patients with implantable cardioverter defibrillators investigated the direct effects of fish oil on ventricular tachyarrhythmia. None of the three trials convincingly showed whether or not supplementation with omega-3 PUFA has preventive effects in these patients [102].

In the randomized, double-blind, placebo-controlled Alpha Omega trial (*N* = 4837) [100], patients with previous myocardial infarction, who were receiving state-of-the-art antihypertensive, antithrombotic, and lipid-modifying therapy, were assigned to use 18.8 g/day of margarine containing a combination of EPA plus DHA (corresponding to 226 mg of EPA + 150 mg of DHA) or placebo for 40 months. At the end of the study, there were no statistically significant differences in the incidence of total fatal or nonfatal CV events between the two groups. However, in this trial the beneficial effects of low-dose EPA plus DHA therapy may have been difficult to prove because the patients were receiving state-of-the-art clinical care [103].

In the OMEGA trial, patients with acute myocardial infarction (*N* = 3851) received 1-g capsules containing either 460 mg EPA and 380 mg DHA or placebo daily in addition to guideline-adjusted therapy and were followed for 1 year [101]. Rates for the primary efficacy outcome of sudden cardiac death were 1.5% in both study arms (*p* = 0.84) Differences in secondary end points such as major CV or cerebrovascular events were also statistically similar. Interpretation of the results from the OMEGA trial was limited because the study lacked sufficient statistical power. The sample size and event rates used in the OMEGA study were based on prior studies, but the patient population was receiving considerably improved guideline-adjusted treatment of acute myocardial infarction, and thus the number of sudden death events was lower than expected [104].

Similar results were obtained in the Supplémentation en Folates et Omega-3 (SU.FOL.OM3) clinical study. This study does not support the use of dietary supplements containing *n*-3 PUFAs (600 mg of EPA and DHA at a ratio of 2:1) for prevention of cardiovascular disease in people with a history of ischaemic heart disease or ischaemic stroke [105].

The effects of long-term treatment with Lovaza (1 capsule/day) on CV events was examined in the Outcome Reduction with an Initial Glargine Intervention (ORIGIN) trial in patients with type 2 diabetes mellitus, impaired fasting glucose, or impaired glucose tolerance (*N* = 12,536). The study found that 1 g/day of EPA/DHA did not prevent death or any CV outcomes in this patient population [106].

In the Omega-3 Fatty Acids for Prevention of Post-operative Atrial Fibrillation (OPERA) trial, the effects of perioperative *n*-3 PUFA supplementation (Lovaza) on the occurrence of postoperative atrial fibrillation was assessed in patients who underwent cardiac surgery (*N* = 1516). No significant difference was observed between patients who received the perioperative *n*-3 PUFA supplementation and those who received placebo [107].

With the aim of assessing the role of *n*-3 PUFAs supplementation on CVD, several meta-analyses have been published, but the conclusions are not straightforward. Two recent meta-analyses published in the same year for example ended with different conclusions: one stated that *n*-3 PUFAs are not universally associated with major CV outcomes across patient populations at increased cardiovascular risk [108]; the second indicated that marine *n*-3 PUFAs, when administrated as food or as supplements for at least six months, reduce CV events by 10%, cardiac death by 9% and coronary events by 18%, while showing a trend toward a lower total mortality [109].

In conclusion, further studies are needed to assess the efficacy of *n*-3 PUFAs therapies in the context of current standards of clinical care, in sufficiently large patient populations and at higher doses. The ongoing, prospective, randomized, double-blind Reduction in Cardiovascular Events with EPA-Intervention Trial (REDUCE-IT; NCT01492361) will assess the ability of Vascepa to reduce CV outcomes in high-risk statin-treated patients with hypertriglyceridemia. Moreover, the VITAL study, an ongoing (NCT01169259) placebo-controlled trial powered to examine major cardiovascular events, as well as CVD and stroke individually, may clarify the utility of *n*-3 PUFAs (Lovaza) in primary prevention of CVD [110].

## 4. Fish Proteins and Cardiovascular Risk Factors

Numerous studies, briefly reviewed here below, have demonstrated beneficial effects of fish proteins and fish-derived peptides on CV risk factors, such as lipid disorders and hypertension (Figure 1).

### 4.1. Fish Proteins and Dyslipidemias

In animal studies, proteins from different fish species have been shown to display hypocholesterolemic activity when compared with casein as protein source [111]. The exact mechanisms responsible for the hypocholesterolemic effect of fish proteins have not been fully identified. The amino acid composition of dietary proteins probably influences plasma cholesterol levels. On this respect, dietary proteins with a low ratio of methionine-glycine and lysine-arginine, such as fish proteins, seem to favor a hypocholesterolemic effect [112,113], in contrast with bovine casein, which tends to elevate cholesterol levels and is characterized by a high ratio of methionine-glycine and lysine-arginine [114]. Mechanistic explanations for the hypocholesterolemic effect of fish proteins include increased hepatic LDL receptor expression [115,116]. Additionally, administration of fish protein hydrolysate to rats, led to an increased hepatic cholesterol 7α-hydroxylase expression and higher cholesterol and bile acids fecal content compared with casein-fed animals [117]. In this study, a hypotriglyceridemic effect by fish protein hydrolysate was also observed.

Among the few clinical studies evaluating the hypocholesterolemic effect of fish proteins, consumption of a cod-fish protein supplement by overweight adults had no effect on triglycerides or HDL-cholesterol levels, but significantly decreased serum LDL-cholesterol levels from baseline [118]. Further studies are needed on larger cohorts and for longer time spans, to confirm the hypocholesterolemic effect of fish protein intake.

### 4.2. Fish Proteins and Hypertension

The antihypertensive activity of peptides from different food sources has been demonstrated both in experimental models and in human volunteers [119]. The discovery of antihypertensive peptides from marine organisms started in the early 1990s, when “Katsuobushi”, a Japanese seasoning prepared from bonito, a fish from tuna family, was examined for its potential to inhibit the activity of angiotensin-converting enzyme (ACE). Fujita *et al.* [120] developed a thermolysin hydrolysate from “Katsuobushi”. This hydrolysate was administered, in fermented drinking water with vinegar, to normotensive human subjects and to patients with mild or moderate hypertension in a small-scale clinical trial. The hydrolysate contained the previously described ACE inhibitory peptide LKPNM [121]. A significant decrease of both systolic and diastolic blood pressure was reported [122].

ACE inhibitory peptides have been found in various other fish species, [123]. Among those, a dipeptide from sardine muscle hydrolysate exhibited antihypertensive effect on mild hypertensive subjects [124]. This same dipeptide was also shown to induce vasodilation thorough a moderate blocking activity on voltage-operated Ca^2+^ channels [125]. Potent ACE inhibitory peptides derived from salmon were also found to possess strong antihypertensive effect in spontaneously hypertensive rats (SHR) [126]. Among 8 proline-containing ACE inhibitory dipeptides isolated from fermented anchovy sauce, KP could significantly lower the blood pressure of SHR [127].

Subsequently, there has been growing interest in exploring the possible uses of the fish by-products or the remaining raw materials, so that they can potentially be utilized rather than posing a waste and sustainability problem [128]. As examples, ACE inhibitory peptides have been produced from sea brame scales [129] and Alaskan Pollack skin [130]. Anti-hypertensive peptides were also found in pepsin digests of bonito pyloric appendix. Those peptides, *in vitro*, inhibited by 40% the endothelin-converting enzyme activity, thus potentially reducing endothelin-1 production [131].

Fish-derived bioactive peptides have enormous potential and have been utilized in the production of pharmaceutical products with an active functional role and effect on health; for example, blood pressure lowering capsules have been manufactured that contain Katsuobushi Oligopeptide LKPNM which is converted into its active form (LKP) by digestive enzymes (Vasotensin 120T^TM^ by Metagenics, Aliso Viejo, CA, USA; PeptACE^TM^ Peptides 90 by Natural Factors, Monroe, WA, USA) [132].

### 4.3. Fish Proteins and Other Potential Anti-Atherosclerotic Effects

Fish protein peptides and hydrolysates have been found to exert antioxidant effects, extensively reviewed in two recent papers [133,134].

Dietary cod proteins were shown to improve insulin sensitivity in insulin-resistant patients [135]. Recently, dietary intake of a salmon peptide fraction prevented glucose intolerance, dyslipidemia, and adipose tissue inflammation in obese mice fed a high-fat, high-sucrose diet [136]. These results suggest that fish peptide supplementation may contribute to the prevention of metabolic syndrome and reduce type 2 diabetes and cardiovascular risk.

**Figure 2:**
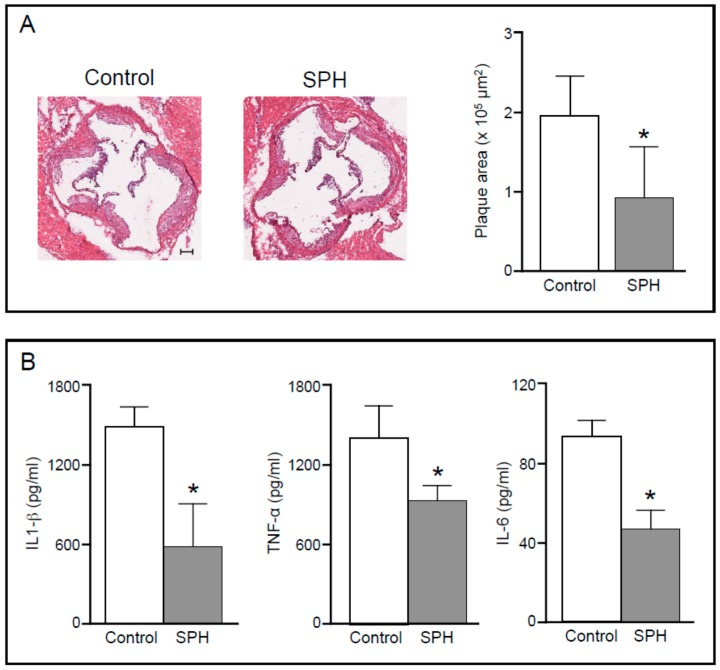
(**A**) Representative photomicrographs (Hematoxilin and Eosin staining) and quantification of maximum plaque area at the aortic sinus in apolipoprotein E deficient mice fed a high-fat diet (Control) or a diet supplemented with 5% salmon protein hydrolysate (SPH) for 12 weeks. * *p* < 0.05 *vs.* Control. Bar = 100 µm; (**B**) Plasma concentrations of IL-1β, TNF-α e IL-6 measured in Control and SPH-treated apolipoprotein E deficient mice at the end of the dietary treatment. * *p* < 0.05 *vs.* Control.

In a recent study, apolipoprotein E deficient mice were fed for 12 weeks a high fat diet containing 21% *w*/*w* casein as protein source (control), or the same diet except for 5% *w*/*w* salmon protein hydrolysate replacing the same amount of casein (SPH) [137]. A significant reduction of atherosclerosis development was observed at the aortic sinus of mice fed SPH compared to controls (Figure 2A). Interestingly, this effect was not accompanied by differences in plasma lipid levels between the two groups, but a significant reduction of circulating pro-inflammatory cytokines was observed in SPH fed mice (Figure 2B), suggesting that salmon protein hydrolysate may reduce atherosclerotic development, at least in part, also by inhibiting activation of systemic inflammation.

## 5. Conclusions

Based on several studies reporting a lower CVD mortality in regular fish consumers, guidelines for healthy individuals encourage the consumption of fish, preferably oily types, at least twice a week. The beneficial effects of fish could be attributed to a wide array of nutrients, particularly *n*-3 PUFAs and proteins/peptides, which display several beneficial effects on the cardiovascular system. The benefit of the isolated nutrients on primary and secondary prevention of CVD has still to be confirmed/proven in further clinical studies.
